# Sleep Deprivation-Induced Changes in Baseline Brain Activity and Vigilant Attention Performance

**DOI:** 10.3390/brainsci12121690

**Published:** 2022-12-09

**Authors:** Maria Paola Tramonti Fantozzi, Tommaso Banfi, Marco Di Galante, Gastone Ciuti, Ugo Faraguna

**Affiliations:** 1 Department of Translational Research and of New Surgical and Medical Technologies, University of Pisa, 56126 Pisa, Italy; 2 The BioRobotics Institute, Scuola Superiore Sant’Anna, 56127 Pisa, Italy; 3 SleepActa s.r.l., 56121 Pisa, Italy; 4 Department of Developmental Neuroscience, IRCCS Fondazione Stella Maris, 56128 Pisa, Italy

**Keywords:** sleep loss, event-related potentials, vigilant attention performance

## Abstract

Sleep deprivation (SD) negatively affects several aspects of cognitive performance, and one of the most widely-used tools to evaluate these effects is the Psychomotor Vigilance Test (PVT). The present study investigated the possibility of predicting changes induced by SD in vigilant attention performance by evaluating the baseline electroencephalographic (EEG) activity immediately preceding the PVT stimuli onset. All participants (*n* = 10) underwent EEG recordings during 10 min of PVT before and after a night of SD. For each participant, the root mean square (RMS) of the baseline EEG signal was evaluated for each 1 s time window, and the respective average value was computed. After SD, participants showed slower (and less accurate) performance in the PVT task. Moreover, a close relationship between the changes in the baseline activity with those in cognitive performance was identified at several electrodes (Fp2, F7, F8, P3, T6, O1, Oz, O2), with the highest predictive power at the occipital derivations. These results indicate that vigilant attention impairments induced by SD can be predicted by the pre-stimulus baseline activity changes.

## 1. Introduction

Human cognitive processes are, in general, analyzed using specific questionnaires and/or computerized cognitive batteries, allowing the acquisition of reliable information about a person’s subjective cognitive performance [1,2,3]. Although their non-intrusiveness, low cost and high validity characteristics, recent studies developed methods using electroencephalogram (EEG) signals for objective and quantitative measurement of cognition [4]. In particular, monitoring brain activity could be useful for quantifying human performance: several EEG analysis methods and signal processing have been applied to evaluate a subject’s performance, mental workload, fatigue, drowsiness, sleepiness and task engagement [4]. Since sleep deprivation (SD) negatively affects several aspects of cognitive performance [5], different studies investigated the EEG changes related to this phenomenon by (a) power spectral density analysis, reflecting the frequency content of the EEG signal, and (b) by the event-related potentials (ERPs) evaluation. In general, the former analysis is used to estimate changes in specific EEG-frequency bands of interest associated with different aspects of cognitive processes such as memory, attention and consciousness [6], while the latter analysis, through the P200 and the P300 component evaluation, investigates the perception of sensorial input and selective attention, respectively [7,8]. In particular, the ERPs extensively studied in relation to attentional processing are the N100, P200 and P300, identified by their polarity and peak latency. Both the negative N100 and the positive P200 correspond to a sensory response and are considered a measure of perceptual encoding processes. They, reflecting selective and executive attentional processes, are influenced by endogenous attention to relevant stimuli, filtering out irrelevant or distracting information [9,10,11]. They could reflect nonspecific attention-triggering (bottom-up) processes and early (top-down) attentional allocation, respectively [8,12,13,14]. While all relevant stimuli elicit the N100 and P200, rare task-relevant items also evoke the so-called P300, a positive deflection occurring around 300 ms after stimulus onset. This later positive component is considered a reliable marker of cognitive updating, discrimination/conscious evaluation of a specific stimulus and attentional allocation [15,16].

EEG analysis revealed that SD leads to specific alterations in the brain’s electrical activity. In particular, after SD, different studies observed a concurrent decrease in the alpha [17,18,19,20] and an increase in the theta [17,19,21] bands power activity. Moreover, lower amplitude and/or prolonged latency of the P300 EEG component was observed after SD during an appropriate auditory or visual cognitive task [22,23,24,25,26,27]. All these changes correlated with sleepiness [22,28,29], reduced vigilance [30], and cognitive performance impairment [30,31,32,33]. While variations in the P300 waveform are consistent across all these studies, the reported changes observed after SD in the P200 component are inconsistent with each other [25,26,27].

One of the most widely used test to evaluate the effects of SD on a specific sphere of cognition is the Psychomotor Vigilance Test (PVT) [34,35], a simple and reliable [35,36] reaction-time (RT) test able to assess sustained vigilant attention, the component of cognition that is dramatically affected in sleep-deprived subjects [35]. Its performance analysis revealed that SD leads to lapses in attention (RT > 500 ms) and to slowed responses, as well as errors of commission [35]. Few studies recorded EEG signals during the execution of the PVT. They demonstrated that the effects of SD are reflected in the EEG activity during the performance of the PVT. In fact, the increment in RT seems to be associated with the increase in the absolute EEG power (4–20 Hz) [37] and with a significant decrease in the P100 amplitude and in the delta and theta phase-locking index [38]. The present study intended to investigate, as a first analysis, the effects of SD on neural mechanisms involved in stimulus processing during a PVT task by the measurement of the phasic EEG activity (N100 and P200 ERPs analysis) and the PVT performance. The second analysis, looking at different topographical areas, investigated the possibility of predicting changes induced by SD in attention abilities by evaluating the corresponding differences in brain activity characteristic of the baseline time window of 1 s immediately preceding stimulus onset. Since the detrimental effects of SD on alertness seem to be related to alterations in the underlying brain physiology, one could expect that sleep loss might induce changes in both tonic (event-unrelated) and phasic (event-related) EEG activity. Furthermore, we hypothesized that these changes could be strictly associated with the deterioration of vigilant attention performance, as assessed by the PVT.

## 2. Materials and Methods

### 2.1. Participants

Experiments were carried out in 10 voluntary healthy participants (age: 24.30 ± 3.68; females (*n* = 4): 22.75 ± 3.30; males (*n* = 6): 25.33 ± 3.83), not affected by neurological, psychiatric, metabolic, or endocrine diseases. At the time of the study, they were not taking any pharmacological drugs. Nine participants were right-handed, while no information was available for the other one.

### 2.2. Experimental Methods

Participants arrived at the sleep laboratory around 7:00 p.m. and were constantly observed throughout the night by one experimenter who made sure they were awake during the entire course of the experiment. In fact, during the entire experiment time, participants, equipped with portable polysomnography to objectively and constantly monitor sleep and brain activity, were prevented from falling asleep and were allowed to engage in their preferred activities. Consumption of any stimulant drugs (such as caffeine) or alcohol was forbidden. To investigate physiological correlates of SD, EEG was recorded while participants performed the PVT, pre- and post-SD, at 8:30 p.m. and at 8:30 a.m. of the next morning, respectively. Only during the pre-SD session, to minimize practice effects, were participants familiarized with a truncated version of the PVT (2 min) and soon after, they were engaged in the first actual test. Moreover, the EEG signal was visually scored by a trained technician to verify the effective sleep deprivation throughout the experiment duration. Sleep staging was computed through Alice Sleepware software: each 30 s EEG epoch was assigned to N1, N2, N3, REM or wakefulness.

### 2.3. Psychomotor Vigilance Task (PVT)

The PVT is a reaction time test used to measure sustained attention. In this task, participants were seated in a comfortable armchair in front of a computer monitor located in the laboratory. Participants had to respond quickly to a visual stimulus presented on the screen at random inter-stimulus intervals of 2–10 s pressing a button on the mouse. The stimulus was a white millisecond timer presented on a black background. After the participant’s response, the RT was displayed on the screen for 1 s. Responses without stimulus presentation or with RT > 500 ms were considered by the system as false starts or lapses of attention, respectively. The task, composed of almost 100 trials, had a duration of about 10 min [39].

### 2.4. Electrophysiological Recordings and EEG Data Analysis

EEG data (sampling rate: 512 Hz) were acquired from 20 gold-plated cup electrodes (Fp1, Fpz, Fp2, F7, F3, Fz, F4, F8, T3, C3, C4, T4, T5, P3, P4, T6, O1, Oz, O2), filled with EC2 paste (EC2^®^ Genuine Grass Electrode Cream, Natus Manufacturing Ltd., Gort, Co. Galway, Ireland), taped and positioned on the scalp according to the 10–20 International Electrodes Placement System. The reference was placed on Cz. EEG data were digitally band-pass filtered (0.5–30 Hz) and were analyzed via EEGLAB toolbox [40] and Matlab R2017b, by custom scripts.

### 2.5. Event-Related Potentials

For the analysis of the event-related potentials, the EEG recordings were time-locked to the onset of the stimuli, and the EEG signal was segmented into time windows encompassing the stimulus and both the preceding and the subsequent second. The average amplitude computed over the 1 s preceding the stimulus onset was subtracted from each trial. Epochs with prominent artifacts were removed by visual inspection, and Independent Component Analysis (ICA) was applied to remove stereotyped muscle and ocular artifacts, as described elsewhere [40]. Epochs recorded within each session (pre- and post-SD) were separately averaged.

For ERPs analysis, the amplitude and latency of N100 and P200 were extracted from the 19 electrodes for each participant. The amplitudes of the N100 and P200 peaks were automatically identified by local peak detection relative to the following time windows after stimulus onset: N100 (130–210 ms) and P200 (210–350 ms) The time interval between the stimulus onset and the peak defined the latency of each ERP component.

### 2.6. Pre-Stimulus, Baseline Activity

For each participant, the root mean square (RMS) of the one-second time window preceding the stimulus onset (baseline signal) was evaluated for each epoch, and the respective average value was computed. This analysis was computed for the total mean power, i.e., for the entire frequency spectrum.

### 2.7. Power Spectrum Density (PSD) of the Baseline Activity

For pre- and post-SD sessions, all single participants’ trials were pooled together, and the PSD of the baseline EEG data was computed for each EEG channel by the EEGLAB Matlab tool “spectopo” function.

### 2.8. Statistical Analysis

All statistical analyses were performed with Statistical Package for Social Sciences (SPSS, version 20), and the level of statistical significance was set at *p* < 0.05.

Neurobehavioral Performance: The RT assessed in the current study comprises the time interval between the onset of the visual stimulus and the participant’s response. To measure the neurobehavioral performance changes in the PVT task, the mean RT and the number of lapses were evaluated by comparing the pre- and post-SD values by paired *t*-test.

Event-Related Potentials: Analysis was performed on N100 and P200 peak amplitudes and latencies. A paired *t*-test was used to investigate the changes in the analogous peak features between pre- and post-SD [41].

Pre-stimulus, Baseline Activity: For each analyzed electrode, a paired sample *t*-test was used to compare the average RMS values obtained pre- and post-SD.

Pre-stimulus, Baseline Activity and Event-Related Potentials/Attention Abilities: The relations between the baseline activity and the N100/P200 peak amplitudes/latencies were tested by linear Pearson’s correlations. Moreover, differences between post-SD with respect to pre-SD values were evaluated for each considered parameter (baseline: ΔRMS; ERPs: ΔN100 Amplitude; ΔN100 Latency; ΔP200 Amplitude; ΔP200 Latency; vigilant attention performance: ΔRT) and correlation analysis was performed between the changes induced by SD in baseline activity and those in ERPs values and attention abilities.

PSD of the Baseline Activity: Average values of the pre- and post-SD spectral power were compared within all the frequency bands, with a resolution of 1 Hz bins, through a paired *t*-test.

When multiple statistical tests were performed, a bootstrap procedure was applied as previously described [42]. As the same size as the original sample (*n* = 10), a new population of participants was performed, allowing repetition. Within this bootstrap sample, pre- and post-SD data were compared by paired *t*-test. This process was repeated 1000 times. For each electrode, the average *p* values and the 5% confidence intervals were computed.

## 3. Results

### 3.1. Descriptive Sleep Data

EEG data scoring showed that, throughout the entire duration of the experiment, participants had brief moments of transition into sleep (total sleep time: 10.7 ± 13.47 min; N1: 5.4 ± 6.71 min; N2: 5.10 ± 7.55 min; N3:0.20 ± 0.63; REM: 0 min).

### 3.2. Vigilant Attention Performance

After SD, participants showed slower (and less accurate) performance in the PVT task. Paired *t*-test comparison of mean RT, number of lapses (RT > 500 ms) and errors in commission between the pre- and post-SD sessions highlighted a significant increment in all these parameters after SD (mean RT: pre-SD: 0.28 ± 0.02 ms; post-SD: 0.36 ± 0.09 ms; *p* = 0.022; t = −2.765; df = 9; number of lapses: pre-SD: 0.70 ± 0.95; post-SD: 8.20 ± 9.95; *p* = 0.033; t = −2.521; df = 9; errors in commission, number of responses without a stimulus: pre-SD: 1.30 ± 1.25; post-SD: 3.40 ± 2.22; *p* = 0.001; t = −4.583; df = 9). These significances were confirmed by the bootstrap procedure.

### 3.3. Pre-Stimulus, Baseline Activity

**Figure 1:**
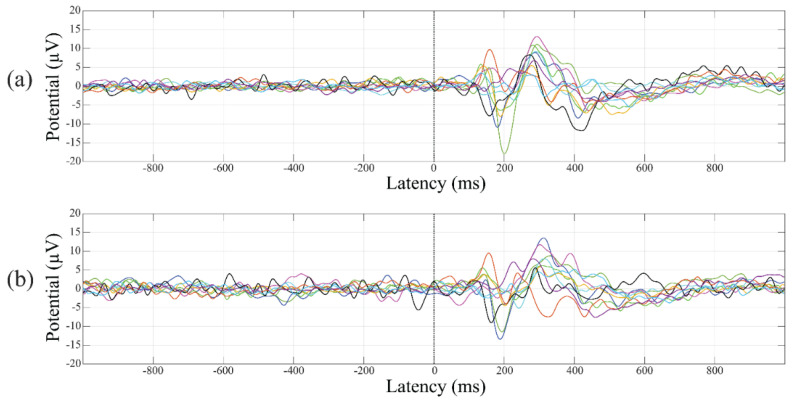
O2, baseline activity in for pre- and post-SD sessions. The average O2 time course of EEG activity evaluated in pre- (**a**) and post- (**b**) SD sessions for each participant analyzed have been superimposed before and after the stimulus onset. Individual participants are represented by lines of different colors. The color code is the same in (**a**,**b**).

Although all electrodes showed an increase in the RMS values of the baseline signal after SD, *t*-test comparisons showed significant differences only in T5 (pre-SD: 8.50 ± 1.84 µV; post-SD: 9.20 ± 2.26 µV, *p* = 0.024, t = −2.711, df = 9), T6 (pre-SD: 8.16 ± 1.67 µV, post-SD: 9.24 ± 2.42 µV, *p* = 0.030, t = −2.581, df = 9), Oz (pre-SD: 7.30 ± 1.96 µV, post-SD: 8.30 ± 2.24 µV, *p* = 0.007, t = −3.489, df = 9) and O2 (pre-SD: 7.39 ± 1.91 µV, post-SD: 8.69 ± 2.43 µV, *p* = 0.003, t = −4.083, df = 9) (Figure 1). Following the bootstrap procedure, the significance was maintained at all these electrodes, with the only exception of T5 (*p* = 0.060).

### 3.4. Pre-Stimulus, Baseline Activity and Behavioral Alertness

**Figure 2:**
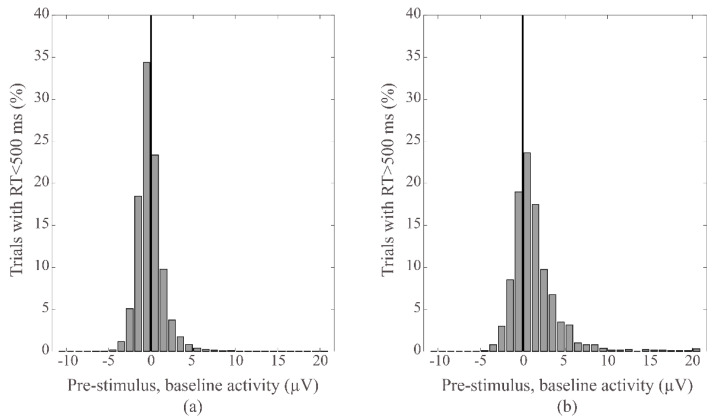
Data distribution of the normalized baseline activity preceding normal responses (**a**); RT < 500 ms; *n* = 26980, mean ± SD: −0.07 ± 1.81 and lapses (**b**); RT > 500 ms; *n* = 1406, mean ± SD: 1.32 ± 2.85; *p* < 0.0005. The continuous vertical line shows the 0 µV. These graphs highlight how the mode of baseline activity preceding lapses is above the 0 µV vertical line (**b**), while the mode for the normal responses remains below (**a**).

For each electrode, all single participants’ trials were pooled together, and the baseline values were normalized to the average value of the corresponding participant. In each electrode, independent *t*-test comparisons showed higher pre-stimulus baseline activity for those epochs preceding RT > 500 ms than for those with RT < 500 ms (Fpz: *p* = 0.006, t = −2.771, df = 1492 all other electrodes: *p* < 0.0005). This finding is represented in Figure 2, showing the average for all trials.

### 3.5. Event-Related Potentials

**Figure 3:**
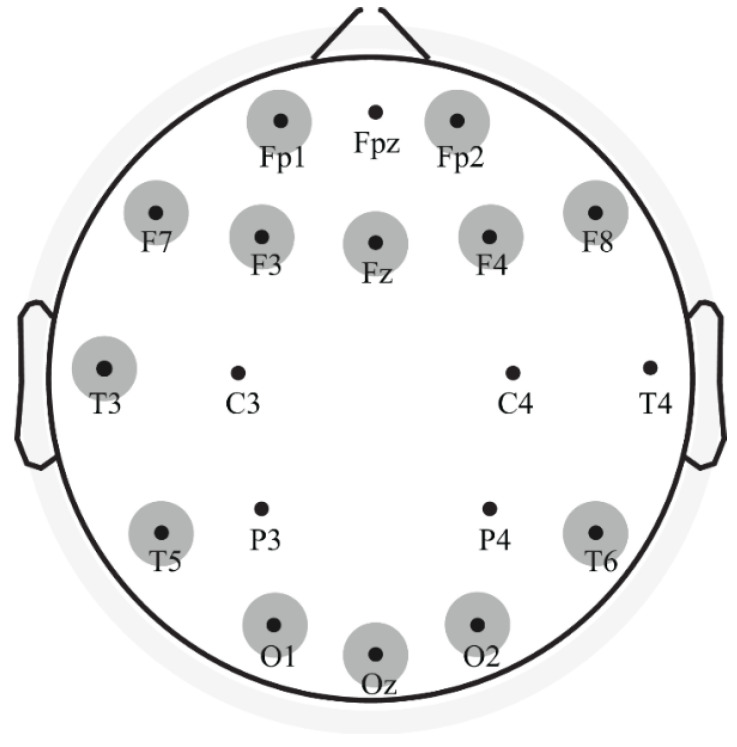
Results of the statistical analysis for the P200 component. The electrodes showing significant differences in latency between pre- and post-SD are indicated by the highlighted circles.

T-test comparisons showed no significant differences in N100 latency and amplitude between pre- and post-SD with the only exception of C3, where SD modified the latency of this component (from 183.60 ± 23.56 to 156.84 ± 26.67 ms, *p* = 0.004, t = 3.783, df = 9). The P200 component showed a post-SD latency increase significant in 13 out of 19 electrodes (Figure 3, Table 1(A)). These results were confirmed by the bootstrap procedure.

No significant differences were found in the P200 amplitude between pre- and post-SD, although a trend towards a decrease is present in all analyzed electrodes (Table 1(B)).

**Figure 4:**
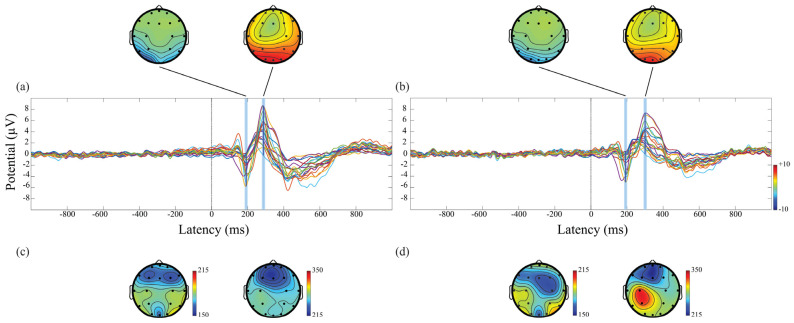
Grand average ERPs evaluated in pre- (**a**) and post- (**b**) sessions for all 19 channels investigated have been superimposed before and after the stimulus. The scalp maps show the topographic distributions of voltage values, color-coded and recorded at the times of N100 and P200 peaks post-stimulus onset. The scalp maps of (**c**,**d**) show the topographic latency of the N100 and P200 peaks, pre- (**c**) and post- (**d**) SD.

Figure 4 shows the ERPs average response of all 19 EEG electrodes, aligned to stimulus onset, recorded before (Figure 4a) and after (Figure 4b) SD, and the corresponding average scalp potential maps obtained at the latencies of N100 and P200. The N100 and the P200 waveforms were spatially consistent across the pre- and the post-SD, with amplitude predominance in the parietal-occipital regions. Regarding the topographic latency of these components (Figure 4c,d), the N100 and the P200 peaks are associated with current flows over frontal (early peaks latency), parietal, and occipital (late peaks latency) scalp areas.

### 3.6. Correlation between Baseline Activity and ERPs Values

Pooling together all electrodes and normalizing the baseline and ERPs values relative to the average value of the corresponding electrode, the baseline activity is negatively correlated with N100 amplitude, pre- (R = −0.309, *p* < 0.0005) and post- (R = −0.345, *p* < 0.0005) SD.

### 3.7. Correlation between Changes in the Baseline Activity and Those in Vigilant Attention Performance

**Figure 5:**
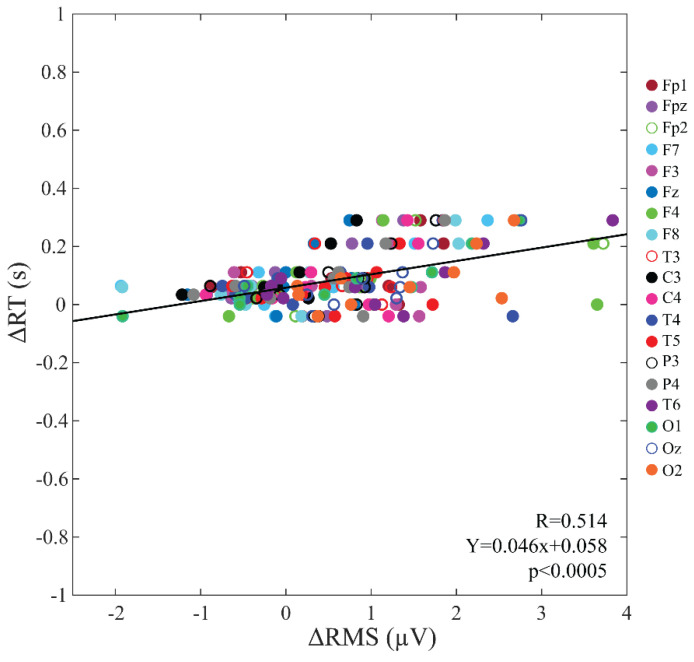
Scatter plot, regression line and corresponding Pearson’s correlation coefficient for the associations between changes induced by SD in the pre-stimulus baseline signal amplitude (RMS) and those in RT. Different electrodes are represented by circles of different colors.

A highly significant positive correlation was found between the changes induced by SD in EEG baseline activity (ΔRMS) and those in vigilant attention performance (ΔRT) (R = 0.514, *p* < 0.0005) (Figure 5).

**Figure 6:**
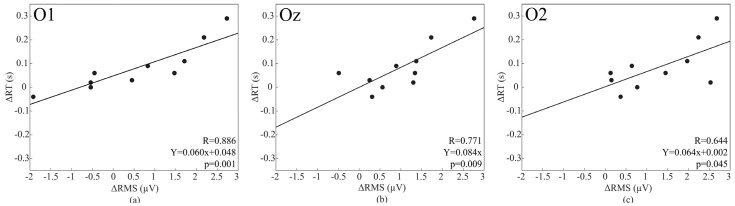
O1 (**a**), Oz (**b**), O2 (**c**): scatter plots, regression lines and corresponding Pearson’s correlation coefficients for the associations between changes induced by SD in the RMS of the baseline signal and those in RT. Circles represent single participant and the continuous lines correspond to the regression line of all the plotted points.

This association was confirmed in 8 out of 19 electrodes (Fp2: R = 0.651, *p* = 0.042; F7: R = 0.741, *p* = 0.014; F8: R = 0.747, *p* = 0.013; P3: R = 0.634, *p* = 0.049; T6: R = 0.736, *p* = 0.015; O1 R = 0.886, *p* = 0.001; Oz: R = 0.771, *p* = 0.009; O2: R = 0.644, *p* = 0.045) and Figure 6 shows the positive correlation at three representative occipital electrodes.

**Figure 7:**
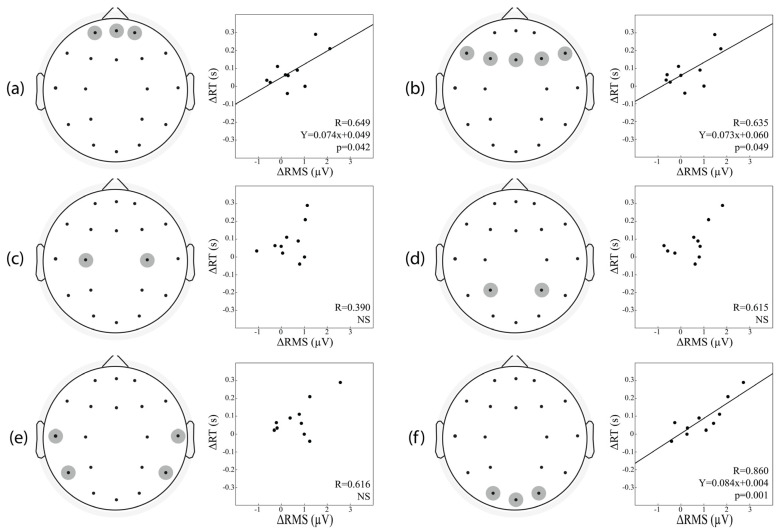
EEG regions of interest (ROIs) for correlational analysis between ΔRMS vs. ΔRT. The EEG electrodes were subdivided into six topographical ROIs according to their location on the scalp obtaining the frontopolar (**a**), frontal (**b**), central (**c**), parietal (**d**), temporal (**e**), and occipital (**f**) area. Within the scatter plots, circles represent single participant and the continuous lines correspond to the regression line of all the plotted points.

In order to further investigate this type of relation, the recorded scalp surface was divided into six regions of interest (ROIs): frontopolar (Fp1, Fp2, Fpz), frontal (F3, F4, F7, F8, Fz), central (C3, C4), parietal (P3, P4), temporal (T3, T4, T5, T6), and occipital (O1, O2, Oz) areas. For each ROI identified, average values of ΔRMS for the corresponding electrodes were evaluated. As shown in Figure 7, a positive correlation was found between ΔRMS and ΔRT in the frontopolar, frontal and occipital ROIs. To better evaluate this relation, a regression model with two independent variables (ΔRMS and ΔERPs values) was applied to ΔRT. This analysis revealed how ΔRT is a better predictor of performance when considering not only the changes in the baseline activity but also those in P200 Amplitude fed simultaneously in a multiple correlation model (Table 2).

### 3.8. PSD of the Baseline Activity

PSD analysis of the baseline activity shows that the amplitude of EEG power spectra, including delta, theta, alpha beta and gamma frequency bands, significantly increased after SD (Figure S1).

## 4. Discussion

Our results, investigating the relationship between EEG and PVT performance, revealed that SD mainly induced: (1) the participant’s vigilant attention impairment, (2) a significant increment in the P200 latency and (3) an increase in the amplitude of EEG baseline brain activity, highly significant at the occipital derivations. This increase was strongly correlated with the detrimental effects of SD on PVT reaction times.

### 4.1. Vigilant Attention Impairment

Our results confirm the susceptibility of PVT performance to SD since all the considered PVT measures (mean RT, number of lapses and errors in commission) were negatively affected by SD. This observation is in line with the knowledge that prolonged wakefulness or lack of sleep are both associated with cognitive deficits [20], probably due to impairments in the cerebral mechanisms mediating cognitive functions [32].

### 4.2. Event-Related Potentials Changes

In agreement with Peng and colleagues [27] and our expectation, we found a significant increment after SD in the latency of the P200 wave (which reflects stimulus evaluation) [43,44], suggesting a lower speed at which the visual PVT stimuli were evaluated and categorized by the sleep-deprived subjects [45]. This lower speed leads to an impairment in attention allocation [6]: indeed, sleep-deprived participants enrolled in this study performed the PVT task with slower responses (increment in RT) and with an increased number of lapses. In pathological conditions such as attention deficit hyper-activity disorder (ADHD) in children [46], Alzheimer’s [47] and Parkinson’s [48] diseases, the higher P200 latency is considered objective evidence of cognitive impairment, indicating dysfunctions in the task-relevant stimuli discrimination. This hypothesis could explain the greater latencies detected in sleep-deprived participants: minimal deficits of the cortical–cortical or cortical–subcortical neural connections could affect information processing with negative effects on cognitive functions [49]. Moreover, in the present experiment, the P200 amplitude tended to decrease following SD in all analyzed electrodes (pre-SD: 5.48 ± 2.22; post-SD: 4.93 ± 2.19; *p* < 0.0005), confirming possible alteration in the information processing after prolonged wakefulness [26].

Additionally, somatosensory N100 component responses have shorter latencies in C3. This finding could be related to hyperexcitability of the motor cortex after SD, as previously described [50,51], probably due to a lack of the brain restorative processes occurring during sleep [50]. In particular, by Transcranial Magnetic Stimulation (TMS) studies, SD seems to induce a reduction of the central motor inhibition [50,51,52,53,54] and of the silent period duration [50], the momentary interruption of a muscle’s electromyographic signal after a motor-evoked potential induced by TMS and a reliable index of the intra-cortical inhibition during voluntary muscle contraction [55]. Since the PVT performance is characterized by both cognitive and sensorimotor components and our participants were mainly right-handed, the N100 shorter latency in C3 after SD could be related to the different impact of sleep loss on the two hemispheres, leading to higher hyperexcitability on the left hemisphere which is the dominant one for our sample.

### 4.3. Baseline Activity Changes

EEG results demonstrate that following SD, the baseline activity tends to increase with detrimental effects on the participant’s alertness. In fact, a close relationship between the (post-pre-SD) changes in the baseline activity with those in vigilant attention performance was identified in several electrodes and especially in the brain region processing visual information (occipital area) where the most significant changes in the baseline activity were identified.

Animal studies showed that, after a sustained period of wakefulness, most cortical neurons respond to perturbation stronger and more synchronously than in the control condition [56], signs of cortical hyperexcitability associated with synaptic potentiation [57,58].

Magnetic resonance imaging (MRI or fMRI) and TMS studies highlighted in young adults, after SD, higher frontal and parietal lobes excitability during a specific cognitive task [54,59,60,61], a phenomenon that correlated with subjective sleepiness. In line with another study [61], our results suggest that SD-induced baseline changes, characteristic of the period immediately pre-stimulus onset, could be considered as a predictor of vigilant attention performance modifications. This could be related to the changes in the subject’s perception [61], alertness and responsiveness to external stimuli that develop together with baseline fluctuations. The increments in cortical synchronization and bistability after SD lead to less neuronal responsiveness to incoming inputs with consequent cognitive impairments [56].

Although no significant relations were observed between changes in baseline activity and those in ERPs values, significant correlations were observed, in line with Lee and colleagues (2011) [62], between the pre-stimulus activity and N100 amplitude, in all sessions. This data confirms the close relationship between the pre-stimulus, baseline activity and the N100 amplitude in the event-related potential study and its associations with performance. Lapse trails were, indeed, characterized by higher pre-stimulus baseline activity.

The relation between post versus pre-SD changes in the pre-stimulus, baseline activity and those in vigilant attention performance could be influenced by changes in P200 amplitude: by multiple regression model we observed that the occipital area of the brain seems to be the best region to predict ΔRT considering changes in both P200 amplitude and baseline activity.

In conclusion, the results of this study indicate that cognitive impairments induced by SD can be predicted by looking at the occipital pre-stimulus baseline activity changes. This finding could pave the way to innovative approaches in the detection of SD-related impairments: the evaluation of the occipital baseline activity during sleep loss could be a simple and reliable non-invasive method useful to track the degree of vigilant attention impairment. Nevertheless, a limitation of the current study is represented by not having been able to detect single-trials features capable of predicting behavioral outcomes. This was the initial aim of the project, with the objective of developing a real-time correction that would, in turn, prevent SD-related behavioral impairments. Most likely, a larger sample of both participants and stimuli would better allow the gain of a reasonable sample size to highlight single-trial differences affecting lapses, setting the ground for real-time intervention. Moreover, further investigations are needed to explore, especially at the occipital electrodes, the SD-induced changes in the frequency components of the baseline EEG signals. Since several studies of quantitative EEG analysis revealed a significant tonic increase in delta and theta power activity after SD [32,51,63,64], where theta power significantly correlated with subjective sleepiness and PVT reaction time performance [32], we may suppose that the higher EEG baseline brain activity amplitude observed in our study could be related to a theta activity increase, with detrimental effects on cognitive performance [31]. We formally tested this hypothesis by computing the average spectrum before and after SD for all subjects. The result shows that the amplitude of EEG power spectra significantly increased after SD across all the frequency bands.

## Figures and Tables

**Table 1:** Mean ± SD of the P200 peak latency and amplitude in pre- and post- SD.

	A			B		
Electrodes	A1. P200 Latency Pre-SD (ms)	A2. P200 Latency Post-SD (ms)	A1vs. A2 *p*=	B1. P200 Amplitude Pre-SD (µV)	B2. P200 Amplitude Post-SD (µV)	B1vs. B2 *p*=
Fp1	252.15 ± 33.23	291.99 ± 45.01	0.003	3.98 ± 3.32	3.80 ± 2.48	0.786
Fpz	249.03 ± 29.48	261.52 ± 41.77	0.252	3.75 ± 2.97	3.16 ± 2.33	0.351
Fp2	253.91 ± 30.17	280.86 ± 40.16	0.003	3.98 ± 3.40	3.35 ± 2.44	0.437
F7	260.55 ± 38.70	309.37 ± 46.99	0.011	5.81 ± 3.60	4.92 ± 2.14	0.330
F3	244.53 ± 24.60	254.49 ± 25.00	0.011	3.29 ± 1.73	2.57 ± 1.55	0.160
Fz	234.38 ± 13.56	257.23 ± 35.71	0.025	2.12 ± 2.13	2.02 ± 1.59	0.841
F4	247.27 ± 25.50	273.24 ± 41.70	0.018	4.05 ± 3.01	3.39 ± 2.22	0.415
F8	258.59 ± 41.67	281.64 ± 47.67	0.024	5.71 ± 3.95	4.68 ± 2.95	0.164
C3	302.34 ± 24.55	312.50 ± 34.38	0.464	1.84 ± 1.07	1.70 ± 1.05	0.477
C4	267.77 ± 28.58	283.20 ± 31.96	0.123	3.93 ± 1.74	3.21 ± 1.59	0.130
T3	270.32 ± 38.93	311.53 ± 42.16	0.004	5.90 ± 3.50	5.20 ± 1.90	0.367
T4	281.64 ± 20.12	309.38 ± 36.46	0.056	5.83 ± 2.76	5.26 ± 2.83	0.464
T5	283.01 ± 23.75	314.45 ± 32.75	0.004	7.71 ± 4.59	7.18 ± 2.76	0.531
T6	273.63 ± 24.44	299.42 ± 43.08	0.016	6.78 ± 4.32	6.44 ± 3.70	0.568
P3	294.73 ± 18.29	310.55 ± 40.73	0.267	6.06 ± 2.36	5.62 ± 1.61	0.399
P4	274.22 ± 23.93	291.60 ± 36.28	0.060	6.31 ± 2.43	5.54 ± 1.89	0.118
O1	279.49 ± 20.49	309.96 ± 27.14	0.001	9.21 ± 2.79	8.57 ± 2.53	0.221
Oz	272.66 ± 23.40	293.56 ± 32.30	0.003	9.89 ± 2.21	9.47 ± 3.48	0.659
O2	280.47 ± 13.73	297.66 ± 24.90	0.004	8.10 ± 3.24	7.65 ± 3.05	0.596

The highlighted boxes represent significant differences.

**Table 2:** Pearson’s correlations between ΔRT and ΔRMS (A)/ΔRMS and ΔP200 Amplitude (B) obtained in the six topographical ROIs.

ROIs	A. ΔRMS	B. ΔRMS, ΔP200 Amplitude
Frontopolar Area	R = 0.649 *p* = 0.042	R = 0.845 *p* = 0.013
Frontal Area	R = 0.635 *p* = 0.049	R = 0.839 *p* = 0.014
Central Area	R = 0.390 *p* = 0.266	R = 882 *p* = 0.005
Parietal Area	R = 0.615 *p* = 0.058	R = 0.819 *p* = 0.020
Temporal Area	R = 0.616 *p* = 0.058	R = 0.752 *p* = 0.054
Occipital Area	R = 0.860 *p* = 0.001	R = 0.930 *p* = 0.001

## Data Availability

Data are available at: https://osf.io/a5fp3/.

## Supplementary Materials

The following supporting information can be downloaded at: https://www.mdpi.com/article/10.3390/brainsci12121690/s1, Figure S1: Comparison between pre- and post-SD average values of power spectrum density (PSD). The lower panel shows the statistical significance for each frequency bin (expressed as 1 − *p*). The horizontal dotted red line represents the significance threshold set as 1 − *p* = 0.95 (*p* = 0.05).

## References

[1] Clark I.A., Maguire E.A. (2020). Do Questionnaires Reflect Their Purported Cognitive Functions?. Cognition.

[2] de Oliveira R.S., Trezza B.M., Busse A.L., Jacob Filho W. (2014). Use of Computerized Tests to Assess the Cognitive Impact of Interventions in the Elderly. Dement. Neuropsychol..

[3] Kane R.L., Kay G.G. (1992). Computerized Assessment in Neuropsychology: A Review of Tests and Test Batteries. Neuropsychol. Rev..

[4] Rabbi A.F., Ivanca K., Putnam A.V., Musa A., Thaden C.B., Fazel-Rezai R. (2009). Human Performance Evaluation Based on EEG Signal Analysis: A Prospective Review. Annu. Int. Conf. IEEE Eng. Med. Biol. Soc..

[5] Alhola P., Polo-Kantola P. (2007). Sleep Deprivation: Impact on Cognitive Performance. Neuropsychiatr. Dis. Treat..

[6] Ward L.M. (2003). Synchronous Neural Oscillations and Cognitive Processes. Trends Cogn. Sci..

[7] Patel S.H., Azzam P.N. (2005). Characterization of N200 and P300: Selected Studies of the Event-Related Potential. Int. J. Med. Sci..

[8] Lijffijt M., Lane S.D., Meier S.L., Boutros N.N., Burroughs S., Steinberg J.L., Moeller F.G., Swann A.C. (2009). P50, N100, and P200 Sensory Gating: Relationships with Behavioral Inhibition, Attention, and Working Memory. Psychophysiology.

[9] Hillyard S.A., Hink R.F., Schwent V.L., Picton T.W. (1973). Electrical Signs of Selective Attention in the Human Brain. Science.

[10] Zhao X., Zhou R., Fu L. (2013). Working Memory Updating Function Training Influenced Brain Activity. PLoS ONE.

[11] Lee E.-Y., Cowan N., Vogel E.K., Rolan T., Valle-Inclán F., Hackley S.A. (2010). Visual Working Memory Deficits in Patients with Parkinson’s Disease Are Due to Both Reduced Storage Capacity and Impaired Ability to Filter out Irrelevant Information. Brain.

[12] Näätänen R., Picton T. (1987). The N1 Wave of the Human Electric and Magnetic Response to Sound: A Review and an Analysis of the Component Structure. Psychophysiology.

[13] Perrault N., Picton T.W. (1984). Event-Related Potentials Recorded from the Scalp and Nasopharynx. I. N1 and P2. Electroencephalogr. Clin. Neurophysiol..

[14] Yurgil K.A., Golob E.J. (2013). Cortical Potentials in an Auditory Oddball Task Reflect Individual Differences in Working Memory Capacity. Psychophysiology.

[15] Polich J. (2007). Updating P300: An Integrative Theory of P3a and P3b. Clinical. Neurophysiol..

[16] van Dinteren R., Arns M., Jongsma M.L.A., Kessels R.P.C. (2014). P300 Development across the Lifespan: A Systematic Review and Meta-Analysis. PLoS ONE.

[17] Patat A., Rosenzweig P., Enslen M., Trocherie S., Miget N., Bozon M.-C., Allain H., Gandon J.-M. (2000). Effects of a New Slow Release Formulation of Caffeine on EEG, Psychomotor and Cognitive Functions in Sleep-Deprived Subjects. Hum. Psychopharmacol..

[18] Corsi-Cabrera M., Sánchez A.I., del-Río-Portilla Y., Villanueva Y., Pérez-Garci E. (2003). Effect of 38 h of Total Sleep Deprivation on the Waking EEG in Women: Sex Differences. Int. J. Psychophysiol..

[19] Ferreira C., Deslandes A., Moraes H., Cagy M., Pompeu F., Basile L.F., Piedade R., Ribeiro P. (2006). Electroencephalographic Changes after One Night of Sleep Deprivation. Arq. Neuropsiquiatr..

[20] Wu J., Zhou Q., Li J., Chen Y., Shao S., Xiao Y. (2021). Decreased Resting-State Alpha-Band Activation and Functional Connectivity after Sleep Deprivation. Sci. Rep..

[21] Hung C.-S., Sarasso S., Ferrarelli F., Riedner B., Ghilardi M.F., Cirelli C., Tononi G. (2013). Local Experience-Dependent Changes in the Wake EEG after Prolonged Wakefulness. Sleep.

[22] Lee H.-J., Kim L., Kim Y.-K., Suh K.-Y., Han J., Park M.-K., Park K.-W., Lee D.-H. (2004). Auditory Event-Related Potentials and Psychological Changes during Sleep Deprivation. Neuropsychobiology.

[23] Gosselin A., De Koninck J., Campbell K.B. (2005). Total Sleep Deprivation and Novelty Processing: Implications for Frontal Lobe Functioning. Clin. Neurophysiol..

[24] Zukerman G., Goldstein A., Babkoff H. (2007). The Effect of 24-40 Hours of Sleep Deprivation on the P300 Response to Auditory Target Stimuli. Aviat. Space Environ. Med..

[25] Ray K., Chatterjee A., Panjwani U., Kumar S., Sahu S., Ghosh S., Thakur L., Anand J.P. (2012). Modafinil Improves Event Related Potentials P300 and Contingent Negative Variation after 24 h Sleep Deprivation. Life Sci..

[26] Zhang L., Shao Y., Liu Z., Li C., Chen Y., Zhou Q. (2019). Decreased Information Replacement of Working Memory After Sleep Deprivation: Evidence From an Event-Related Potential Study. Front. Neurosci..

[27] Peng Z., Dai C., Ba Y., Zhang L., Shao Y., Tian J. (2020). Effect of Sleep Deprivation on the Working Memory-Related N2-P3 Components of the Event-Related Potential Waveform. Front. Neurosci..

[28] Strijkstra A.M., Beersma D.G.M., Drayer B., Halbesma N., Daan S. (2003). Subjective Sleepiness Correlates Negatively with Global Alpha (8-12 Hz) and Positively with Central Frontal Theta (4–8 Hz) Frequencies in the Human Resting Awake Electroencephalogram. Neurosci. Lett..

[29] Vyazovskiy V.V., Tobler I. (2005). Theta Activity in the Waking EEG Is a Marker of Sleep Propensity in the Rat. Brain Res..

[30] Lee H.-J., Kim L., Suh K.-Y. (2003). Cognitive Deterioration and Changes of P300 during Total Sleep Deprivation. Psychiatry Clin. Neurosci..

[31] Klimesch W. (1999). EEG Alpha and Theta Oscillations Reflect Cognitive and Memory Performance: A Review and Analysis. Brain Res. Rev..

[32] Gorgoni M., Ferlazzo F., Ferrara M., Moroni F., D’Atri A., Fanelli S., Gizzi Torriglia I., Lauri G., Marzano C., Rossini P.M. (2014). Topographic Electroencephalogram Changes Associated with Psychomotor Vigilance Task Performance after Sleep Deprivation. Sleep Med..

[33] Posada-Quintero H.F., Reljin N., Bolkhovsky J.B., Orjuela-Cañón A.D., Chon K.H. (2019). Brain Activity Correlates With Cognitive Performance Deterioration During Sleep Deprivation. Front. Neurosci..

[34] Dinges D.F., Powell J.W. (1985). Microcomputer Analyses of Performance on a Portable, Simple Visual RT Task during Sustained Operations. Behav. Res. Methods Instrum. Comput..

[35] Lim J., Dinges D.F. (2008). Sleep Deprivation and Vigilant Attention. Ann. N. Y. Acad. Sci..

[36] Jewett M.E., Dijk D.J., Kronauer R.E., Dinges D.F. (1999). Dose-Response Relationship between Sleep Duration and Human Psychomotor Vigilance and Subjective Alertness. Sleep.

[37] Corsi-Cabrera M., Arce C., Ramos J., Lorenzo I., Guevara M.A. (1996). Time Course of Reaction Time and EEG While Performing a Vigilance Task during Total Sleep Deprivation. Sleep.

[38] Hoedlmoser K., Griessenberger H., Fellinger R., Freunberger R., Klimesch W., Gruber W., Schabus M. (2011). Event-Related Activity and Phase Locking during a Psychomotor Vigilance Task over the Course of Sleep Deprivation. J. Sleep Res..

[39] Khitrov M.Y., Laxminarayan S., Thorsley D., Ramakrishnan S., Rajaraman S., Wesensten N.J., Reifman J. (2014). PC-PVT: A Platform for Psychomotor Vigilance Task Testing, Analysis, and Prediction. Behav. Res. Methods.

[40] Delorme A., Makeig S. (2004). EEGLAB: An Open Source Toolbox for Analysis of Single-Trial EEG Dynamics Including Independent Component Analysis. J. Neurosci. Methods.

[41] Li F., Yi C., Jiang Y., Liao Y., Si Y., Dai J., Yao D., Zhang Y., Xu P. (2019). Different Contexts in the Oddball Paradigm Induce Distinct Brain Networks in Generating the P300. Front. Hum. Neurosci..

[42] Tramonti Fantozzi M.P., Artoni F., Di Galante M., Briscese L., De Cicco V., Bruschini L., d’Ascanio P., Manzoni D., Faraguna U., Carboncini M.C. (2021). Effect of the Trigeminal Nerve Stimulation on Auditory Event-Related Potentials. Cereb. Cortex Commun..

[43] Saito H., Yamazaki H., Matsuoka H., Matsumoto K., Numachi Y., Yoshida S., Ueno T., Sato M. (2001). Visual Event-Related Potential in Mild Dementia of the Alzheimer’s Type. Psychiatry Clin. Neurosci..

[44] Potts G.F. (2004). An ERP Index of Task Relevance Evaluation of Visual Stimuli. Brain Cogn..

[45] Breznitz Z., Meyler A. (2003). Speed of Lower-Level Auditory and Visual Processing as a Basic Factor in Dyslexia: Electrophysiological Evidence. Brain Lang..

[46] Anjana Y., Khaliq F., Vaney N. (2010). Event-Related Potentials Study in Attention Deficit Hyperactivity Disorder. Funct. Neurol..

[47] Martinelli V., Locatelli T., Comi G., Lia C., Alberoni M., Bressi S., Rovaris M., Franceschi M., Canal N. (1996). Pattern Visual Evoked Potential Mapping in Alzheimer’s Disease: Correlations with Visuospatial Impairment. Dementia.

[48] Wascher E., Verleger R., Vieregge P., Jaskowski P., Koch S., Kömpf D. (1997). Responses to Cued Signals in Parkinson’s Disease. Distinguishing between Disorders of Cognition and of Activation. Brain.

[49] Leocani L., Gonzalez-Rosa J.J., Comi G. (2010). Neurophysiological Correlates of Cognitive Disturbances in Multiple Sclerosis. Neurol. Sci..

[50] Scalise A., Desiato M.T., Gigli G.L., Romigi A., Tombini M., Marciani M.G., Izzi F., Placidi F. (2006). Increasing Cortical Excitability: A Possible Explanation for the Proconvulsant Role of Sleep Deprivation. Sleep.

[51] De Gennaro L., Marzano C., Veniero D., Moroni F., Fratello F., Curcio G., Ferrara M., Ferlazzo F., Novelli L., Concetta Pellicciari M. (2007). Neurophysiological Correlates of Sleepiness: A Combined TMS and EEG Study. Neuroimage.

[52] Civardi C., Boccagni C., Vicentini R., Bolamperti L., Tarletti R., Varrasi C., Monaco F., Cantello R. (2001). Cortical Excitability and Sleep Deprivation: A Transcranial Magnetic Stimulation Study. J. Neurol. Neurosurg. Psychiatry.

[53] Kreuzer P., Langguth B., Popp R., Raster R., Busch V., Frank E., Hajak G., Landgrebe M. (2011). Reduced Intra-Cortical Inhibition after Sleep Deprivation: A Transcranial Magnetic Stimulation Study. Neurosci. Lett..

[54] Huber R., Mäki H., Rosanova M., Casarotto S., Canali P., Casali A.G., Tononi G., Massimini M. (2013). Human Cortical Excitability Increases with Time Awake. Cereb. Cortex.

[55] Hupfeld K.E., Swanson C.W., Fling B.W., Seidler R.D. (2020). TMS-Induced Silent Periods: A Review of Methods and Call for Consistency. J. Neurosci. Methods.

[56] Vyazovskiy V.V., Olcese U., Cirelli C., Tononi G. (2013). Prolonged Wakefulness Alters Neuronal Responsiveness to Local Electrical Stimulation of the Neocortex in Awake Rats. J. Sleep Res..

[57] Bliss T.V., Lomo T. (1973). Long-Lasting Potentiation of Synaptic Transmission in the Dentate Area of the Anaesthetized Rabbit Following Stimulation of the Perforant Path. J. Physiol..

[58] Vyazovskiy V.V., Cirelli C., Pfister-Genskow M., Faraguna U., Tononi G. (2008). Molecular and Electrophysiological Evidence for Net Synaptic Potentiation in Wake and Depression in Sleep. Nat. Neurosci..

[59] Drummond S.P., Brown G.G., Gillin J.C., Stricker J.L., Wong E.C., Buxton R.B. (2000). Altered Brain Response to Verbal Learning Following Sleep Deprivation. Nature.

[60] Chee M.W.L., Choo W.C. (2004). Functional Imaging of Working Memory after 24 Hr of Total Sleep Deprivation. J. Neurosci..

[61] Boly M., Balteau E., Schnakers C., Degueldre C., Moonen G., Luxen A., Phillips C., Peigneux P., Maquet P., Laureys S. (2007). Baseline Brain Activity Fluctuations Predict Somatosensory Perception in Humans. Proc. Natl. Acad. Sci. USA.

[62] Lee T.-W., Yu Y.W.-Y., Wu H.-C., Chen T.-J. (2011). Do Resting Brain Dynamics Predict Oddball Evoked-Potential?. BMC Neurosci..

[63] Cajochen C., Brunner D.P., Kräuchi K., Graw P., Wirz-Justice A. (1995). Power Density in Theta/Alpha Frequencies of the Waking EEG Progressively Increases during Sustained Wakefulness. Sleep.

[64] Aeschbach D., Matthews J.R., Postolache T.T., Jackson M.A., Giesen H.A., Wehr T.A. (1997). Dynamics of the Human EEG during Prolonged Wakefulness: Evidence for Frequency-Specific Circadian and Homeostatic Influences. Neurosci. Lett..

