# Supplementary material for: Sleep Deprivation-Induced Changes in Baseline Brain Activity and Vigilant Attention Performance
Source: Brain Sci. 2022 Dec 9;12(12):1690. doi: 10.3390/brainsci12121690 (PMC9775863; doi:10.3390/brainsci12121690)
Supplement: Supplementary file 1 [file brainsci-12-01690-s001.zip › brainsci-2044620-supplementary.pdf]

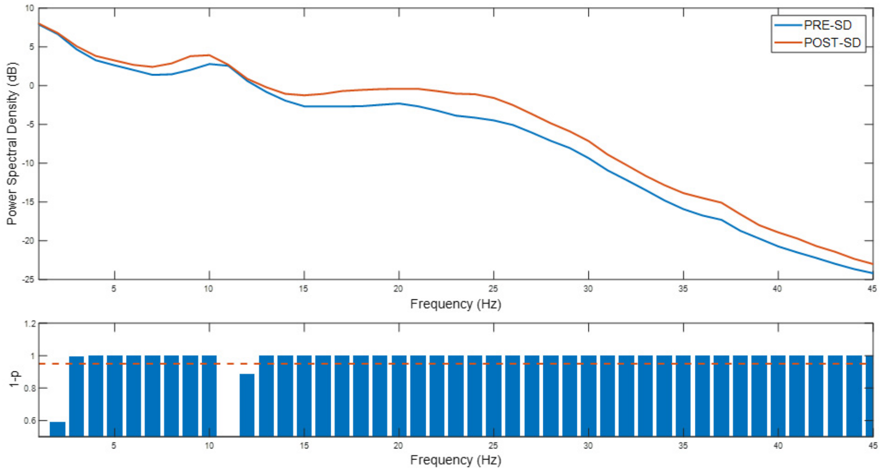

Figure S1: Comparison between pre- and post-SD average values of power spectrum density (PSD). The lower panel shows the statistical significance for each frequency bin (expressed as $1 - p$). The horizontal dotted red line represents the significance threshold set as $1 - p = 0.95$ ($p = 0.05$).
